# Influence of Global Spine Sagittal Balance and Spinal Degenerative Changes on Locomotive Syndrome Risk in a Middle-Age and Elderly Community-Living Population

**DOI:** 10.1155/2020/3274864

**Published:** 2020-09-23

**Authors:** Masaaki Machino, Kei Ando, Kazuyoshi Kobayashi, Hiroaki Nakashima, Shunsuke Kanbara, Sadayuki Ito, Taro Inoue, Hidetoshi Yamaguchi, Hiroyuki Koshimizu, Taisuke Seki, Shinya Ishizuka, Yasuhiko Takegami, Naoki Ishiguro, Yukiharu Hasegawa, Shiro Imagama

**Affiliations:** ^1^Department of Orthopaedic Surgery, Nagoya University Graduate School of Medicine, 65 Tsurumai, Showa-ku, Nagoya, Aichi 466-8550, Japan; ^2^Department of Rehabilitation, Kansai University of Welfare Sciences 3-11-1, Asahigaoka, Kashiwara, Osaka 582-0026, Japan

## Abstract

**Purpose:**

The aim of this study was to describe the characteristics of each locomotive syndrome (LS) risk stage, including global spine sagittal alignment, spinal degenerative changes evident on plain radiographs, low back pain (LBP), muscle strength, and physical ability in middle-aged and elderly people in a health checkup.

**Methods:**

This study included 211 healthy Japanese volunteers (89 men and 122 women; mean age, 64.0 years) who underwent assessment with both radiographs and Spinal Mouse. Spinal sagittal parameters included thoracic kyphosis angle (TKA), lumbar lordosis angle (LLA), sagittal vertical axis, and spinal inclination angle (SIA). Lumbar disc height (LDH) and lumbar osteophyte formation (LOF) at each level were evaluated as the spinal degenerative changes. The LS assessment comprised three tests: stand-up test, two-step test, and 25-question Geriatric Locomotive Function Scale (GLFS-25). The subjects were divided into three groups (no risk, stage 1 LS, or stage 2 LS) according to LS risk test criteria. The prevalence of LBP was investigated with a visual analogue scale (VAS), and physical performances were also compared among the groups.

**Results:**

Of the participants, 122 had no risk of LS, 56 had stage 1 LS risk, and 29 had stage 2 LS risk. With increasing LS risk stage, the prevalence of and VAS score for LBP increased significantly, and back muscle strength and physical abilities decreased significantly. The TKA did not differ among the three groups. The LLA decreased gradually with LS risk stage (*P* = 0.0001). At each level except L1–L2 and L5–S1, LDH decreased gradually with LS risk stage. The prevalence of LOF increased significantly with increasing LS risk stage. The SIA increased significantly with LS risk stage (*P* = 0.0167).

**Conclusions:**

Participants with LS had higher prevalence of spinal degeneration, small LLA, and global spinal imbalance by anterior spinal inclination.

## 1. Introduction

Human locomotion is a fundamental activity that greatly affects quality of life (QOL). With aging, this function declines as a result of the degeneration of various organs. Therefore, for middle-aged persons, it is very important to maintain motor function and prevent a decrease in it for a long and healthy life. In 2007, the Japanese Orthopaedic Association (JOA) proposed the concept of “locomotive syndrome” (LS) as a condition in people with musculoskeletal disease who are highly likely to require future nursing care [1, 2]. In advanced stages, LS directly affects the quality and activities of daily living (ADL) [3–5]. Furthermore, in addition to a significantly poorer QOL, the risk for falling is increased in the elderly population with LS [6, 7]. Prevention of LS has long been advocated for maintaining and improving motor function of middle-aged and elderly people [8–10]. According to methods for evaluating the LS risk, LS is categorized into three stages [11]: at stage 1, interventions such as exercise training are needed; at stage 2, the patient requires a medical checkup to determine the underlying pathologic process for the problem to be resolved.

Relative factors for decreased QOL include spinal kyphosis, chronic pain, poor muscle strength, and poor physical ability [4–9]. The effect of spinal sagittal misalignment, such as kyphosis, on QOL and mortality has been examined [10]. Our previous study demonstrated that the relationship between low back pain (LBP), QOL, and LS risk in middle-aged and elderly people [11]. However, there has been no analysis of spinal sagittal alignment and spinal degeneration in each LS risk stage in the healthy general population. Therefore, a new cohort study was conducted to investigate the relationship between spinal alignment, degeneration, and LS risk during another survey period. The aim of this study was to describe the characteristics of each LS risk stage, including global spine sagittal alignment, spinal degenerative changes evident on plain radiographs, as well as LBP, muscle strength, and physical ability in middle-aged and elderly people in a health checkup.

## 2. Methods

### 2.1. Subjects

The subjects were healthy Japanese volunteers who attended a basic health checkup supported by the local government in 2018. This checkup, held annually in the town of Yakumo in a rural area of southern Hokkaido, Japan, comprises voluntary orthopedic, physical function, and internal medical examinations [12]. This study included patients who had undergone radiographic assessment and motor function testing and had completed a self-report questionnaire. Exclusion criteria were as follows: history of spinal or knee surgery, fresh vertebral fracture, insufficient quality of radiograph, and insufficient physical examination data. The prevalence of LBP and sciatica were investigated with a visual analogue scale (0 to 100 mm, VAS), as in previous studies [13].

Two hundred eighty-two subjects underwent the physical performance test, the LS risk test, radiography of the whole spine, and the bone status examination. Of these 282 subjects, the 211 (89 men and 122 women; mean age, 64.0 years) who underwent Spinal Mouse® (Idiag, Volkerswill, Switzerland) measurements were included in the final study population. Spinal Mouse is a computer-assisted device with which spinal shape and mobility is assessed with surface-based measurements. A previous cohort study showed that this method can be used to evaluate these parameters without invasive irradiation [5, 8, 10]. The study protocol was approved by the ethics committee in human research and the institutional review board of our university. All subjects provided written informed consent. The study procedures were carried out in accordance with the principles of the Declaration of Helsinki.

### 2.2. Physical Performance

Back muscle strength, as the maximal isometric strength of the trunk muscles in a standing posture with 30° lumbar flexion, was measured one time with a digital back muscle strength meter (T.K.K.5402; Takei Scientific Instruments Co., Niigata, Japan) [4]. Subjects walked a straight 10-m course one time at their fastest pace, and the time necessary to complete the course was recorded as the 10-m gait time [4, 5, 11]. In the 3-m timed up-and-go test (3-m TUG), subjects rose from a standard chair (46-cm seat height from the ground), walked a distance of 3 m, turned around, walked back to the chair, and sat down, and the time necessary to accomplish this was measured. The mean of two 3-m TUG trials was recorded. The maximum stride length was measured, while subjects were in a standing position; subjects put their right foot forward as far as they could and then brought the left foot up to the right foot without touching the floor with their hands or knees. This was repeated with the left foot forward; the average value of the two distances was divided by the subject's height, and the result was used as the maximum stride length [8, 9, 14].

### 2.3. Locomotive Syndrome Risk Test

The LS risk test consists of three parts: a stand-up test, a two-step test, and the 25-question Geriatric Locomotive Function Scale (GLFS-25). These three tests were performed in the same way as described in previous studies [11, 15, 16]. The JOA defines two stages in the LS risk test. LS risk stage 1 is defined as a two-step test score of less than 1.3, difficulty with standing from a 40-cm-high seat in the stand-up test with one leg (either leg), or a 25-question GLFS score of 7 or higher; subjects meeting any of these criteria received a diagnosis of a beginning decline in mobility. LS risk stage 2 is defined as a two-step test score of less than 1.1, difficulty with rising from a 20-cm-high seat with the use of both legs in the stand-up test, or a 25-question GLFS score of 16 or higher; subjects meeting any of these criteria received a diagnosis of progression of decline in mobility. In this study, subjects who met the criteria for LS risk test stage 1 or 2 were defined as “LS risk subjects,” and the other subjects were “no risk subjects”.

#### 2.3.1. Stand-Up Test

The stand-up test is used to evaluate the range of motion at the joint, flexibility, and balance, in addition to lower extremity muscle strength [11, 15, 16]. Difficulty in one-leg rising (either leg) from a 40-cm-high seat is classified as stage 1 LS risk. Difficulty in rising from a 20-cm-high seat with the use of both legs is classified as stage 2 LS risk.

#### 2.3.2. Two-Step Test

The two-step test was previously reported by Muranaga and Hirano and has been developed as a screening tool for fundamental walking ability. In the two-step test, subjects stood with their toes behind a starting line, took two steps that were as long as possible, and then aligned both feet. The two-step test score was obtained as the length of the two steps (in centimeters) divided by height (in centimeters). A score between 1.1 and 1.3 points is classified as stage 1 LS risk, and a score of less than 1.1 points is classified as stage 2 LS risk [11, 15, 16].

#### 2.3.3. Self-Report 25-Question Geriatric Locomotive Function Scale

The GLFS-25 is a self-administered questionnaire, consisting of 25 items graded on a 5-point scale, ranging from no impairment (0 points) to severe impairment (4 points). The sum of these scores is the total possible score, which ranges between 0 and 100; increasing values indicate increasing severity of LS [16–18]. This measurement has been validated as evaluating activities of daily life for the Japanese elderly population and for elderly people of other races. The GLFS-25 has a high internal consistency value, excellent reproducibility, and high interobserver and intraobserver values. A GLFS score between 7 and 15 points indicates grade 1 LS risk, and a score>16 points indicates stage 2 LS risk.

### 2.4. Measurement of Spinal Alignment

Free-standing spine radiographs with fists on clavicles were obtained in all subjects. All images were transferred to a computer as Digital Imaging and Communications in Medicine (DICOM) data. For each subject, the spinal parameter was obtained in lateral spine radiographs: thoracic kyphosis (affecting T4–L1, TK), lumbar lordosis (affecting L1–S1, LL), and sagittal vertical axis (affecting C7–S1, SVA) were assessed with measurement software. Lumbar disc height (LDH) and lumbar osteophyte formation (LOF) were investigated from L1–L2 level to L5–S1 level, to evaluate the spinal degenerative changes. LOF was evaluated using the Nathan classification (0–4) at each segment [19], as reported in our previous study [20], with positive osteophyte formation defined as class 2 in this study. The prevalence of LOF was compared among the three groups of subjects (no risk, stage 1 LS risk, and stage 2 LS risk). Each parameter was measured by two experienced spinal surgeons who used the Surgimap, version 2.2.9.6 (Surgimap Spine Software, Nemaris Inc., New York, NY, USA). Surgimap is an open-source archive system and computer software that allows geometrical calculations of alignment [21, 22]. A positive lumbar lordosis angle indicates lordosis in this study. Measurement of spinal curvature was also evaluated with Spinal Mouse. Intraclass coefficients of 0.92 to 0.95 have been determined for curvature measurement with Spinal Mouse [23]. In this study, we measured the angles at neutral standing position. The evaluation items included thoracic kyphosis angle (T1–T12, TKA), lumbar lordosis angle (T12–S1, LLA), and the spinal inclination angle (SIA) between the straight line from T1 to S1 and the true vertical. Spinal inclination was reflected by a forward, stooped posture. All spinal data could be measured and then calculated automatically and easily with the use of the Spinal Mouse apparatus [23, 24].

### 2.5. Statistical Analysis

All data were analyzed using the statistical software SPSS (version 25.0; SPSS Statistics, IBM Corp., Armonk, NY, USA). Results are expressed as means ± standard deviations. For nonparametric analysis, the Mann–Whitney *U* test was used to analyze between-group differences, whereas the Kruskal-Wallis test, followed by the Mann–Whitney *U* test, was used for comparisons among three groups. Spearman's rank correlation coefficient was used to determine correlations. The chi-square test was used to analyze differences between groups. A *P* value of less than 0.05 was considered statistically significant.

## 3. Results

Of the 211 subjects, 122 were classified as having no risk for LS, 56 as having stage 1 LS, and 29 as having stage 2 LS. No differences were found among the three groups with regard to age, sex, body height, body weight, or body mass index (BMI) (Table 1).

The prevalence (%) of LBP increased significantly with increasing LS risk stage (15.9 vs. 32.1 vs. 44.8, for the no risk, stage 1 and stage 2 group, respectively; *P* = 0.0012). The VAS score (mm) for LBP increased significantly with increasing LS risk stage (7.54 ± 14.4 vs. 17.7 ± 19.7 vs. 25.2 ± 23.7, *P* < 0.0001). Although there were no differences in the prevalence of sciatica among the three groups (1.6% vs. 3.6 vs. 6.9%, *P* = 0.2792), the VAS score (mm) of sciatica tended to increase with the LS risk stage (4.13 ± 10.4 vs. 11.8 ± 16.6 vs. 20.4 ± 25.4, *P* < 0.0001).

Back muscle strength (kg) decreased significantly with increasing LS risk stage (83.5 ± 31.9 vs. 76.4 ± 26.1 vs. 66.1 ± 24.7, *P* = 0.0490). The 10-m gait time (s) became significantly longer with increasing LS risk stage (4.50 ± 0.61 vs. 4.89 ± 0.83 vs. 5.14 ± 0.96, *P* < 0.0001). The 3-m TUG (s) also became significantly longer with increasing LS risk stage (5.79 ± 0.76 vs. 6.21 ± 0.91 vs. 6.43 ± 1.01, *P* < 0.0001). The percentage (%) of maximum stride tended to decrease with increasing LS risk stage (80.2 ± 3.53 vs. 74.1 ± 1.29 vs. 70.5 ± 8.83, *P* = 0.0009).

**Figure 1:**
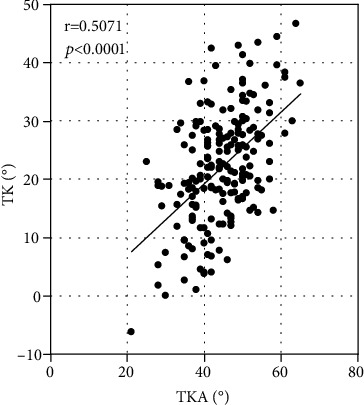
Correlation between thoracic kyphosis (TK) as assessed on radiographs (T4–L1) and thoracic kyphosis angle (TKA) as assessed with Spinal Mouse (T1–T12).

**Figure 2:**
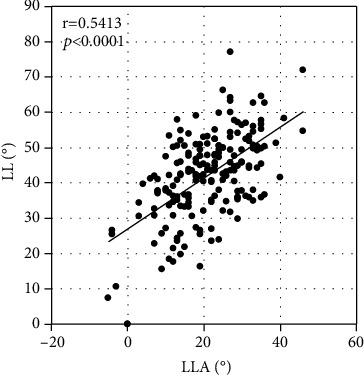
Correlation of lumbar lordosis (LL) as assessed on radiographs (L1–S1) and lumbar lordosis angle (LLA) as assessed with Spinal Mouse (T12–S1).

**Figure 3:**
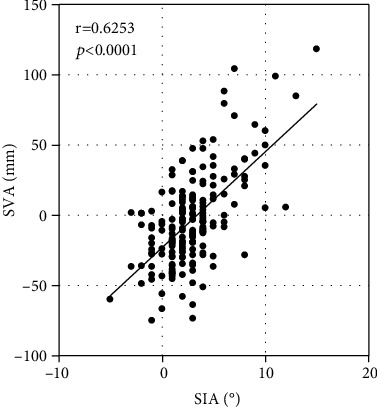
Correlation of the global spine sagittal balance between sagittal vertical axis (SVA) as assessed on radiographs (C7–S1) and spinal inclination angle (SIA) as assessed with Spinal Mouse (T1–S1).

The thoracic kyphosis assessed on radiographs was moderately related to that assessed by Spinal Mouse, according to a correlation coefficient (*r* = 0.5071, *P* < 0.0001; Figure 1). A significant correlation in the lumbar lordosis was observed between radiographs and Spinal Mouse (*r* = 0.5413, *P* < 0.0001) (Figure 2). The global spine sagittal balance significantly correlated between SVA of radiographs and SIA of Spinal Mouse (*r* = 0.6253, *P* < 0.0001). (Figure 3).

**Figure 4:**
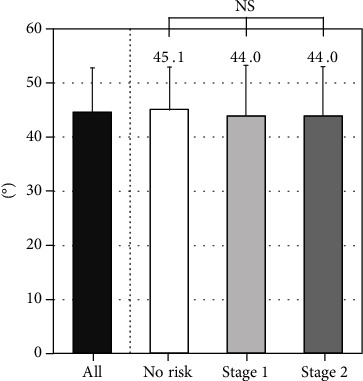
Association between locomotive syndrome risk and thoracic kyphosis angle (TKA).

**Figure 5:**
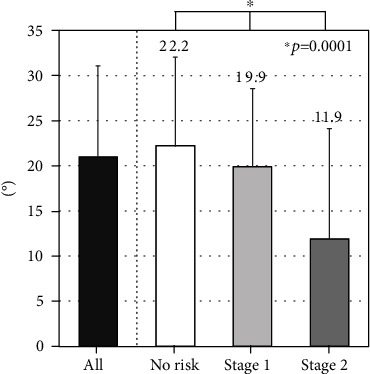
Association between locomotive syndrome risk and lumbar lordosis angle (LLA).

**Figure 6:**
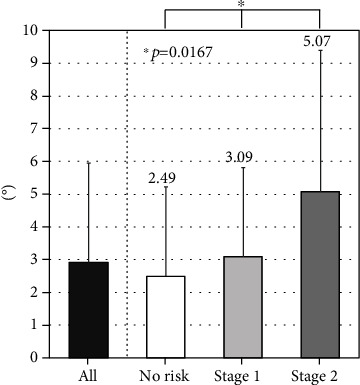
Association between locomotive syndrome risk and spinal inclination angle (SIA).

There were no differences in the TKA among the three groups (Figure 4). The LLA decreased gradually with LS risk stage (*P* = 0.0001; Figure 5). At each level except for L1–L2 and L5–S1, LDH decreased gradually with LS risk stage (Table 2). At all segments, the prevalence of LOF increased significantly with increasing LS risk stage (Table 3). The SIA tended to increase with LS risk stage (*P* = 0.0167). The SIA was highest for subjects with LS risk stage 2 (Figure 6).

## 4. Discussion

This study demonstrated the characteristics of each LS risk stage. Subjects with LS had significantly severe LBP, poorer back muscle strength, and poorer physical ability. This study also revealed a relationship between spinal parameters and LS risk stage. To our knowledge, this is the first study in which three groups (no risk, stage 1 LS, and stage 2 LS) were examined with regard to the relationship between global spine sagittal balance and spinal degenerative changes in a health checkup. Interestingly, lower lumbar lordosis, higher spinal inclination, and higher prevalence of spinal degeneration were all found to be significantly related to LS risk, even though there was no difference in age among the three groups.

Tokida et al. reported that the prevalence of LS and LS risk stage significantly increased with age [25]. In present study, although the age (years) of subjects increased with increasing LS risk stage (62.8 vs. 65.3 vs. 67.0, for the no risk, stage 1, and stage 2 group, respectively), there were no significant differences (*P* = 0.1072). The inclusion criteria in this study were subjects who over 40 years, but the participants between the ages of 50–89 were included in previous study. The age differences of the subjects might have caused a discrepancy with the results of previous study.

As LS develops, affected patients first might have back muscle weakness; then, lumber alignment becomes kyphotic and LOF as a result of decreasing LDH. After that, LBP and global spinal malalignment by anterior spinal inclination occur. Finally, gait speed slows down, and stride becomes narrower. After lumbar kyphotic changes, compensatory changes such as decreased thoracic kyphosis prevent bending forward and maintain spinal sagittal alignment [26]. However, in some elderly people, this compensation does not occur, and anterior spinal inclination (a bent-forward posture) results from lumbar kyphosis [26]. Lumbar kyphosis and anterior spinal inclination cause serious strain on the spine during walking, while the person looks ahead and on ADL, which results in the development of LS and LBP. Conversely, these kinds of pain could possibly cause spinal malalignment, but the subjects in this study were generally healthy without severe pain that might have aggravated spinal posture.

Because of the pivotal role of the spine in motor function, global spine sagittal alignment is essential. In particular, the SIA was reported to be increased in subjects affected with LS [8, 10, 23]. Hirano et al. reported that increased SIA and decreased back muscle strength were significantly related to LS, according to multivariate logistic analysis [8, 27, 28]. They also concluded that lumbar kyphosis is an important factor related to back muscle strength and SIA. Muramoto et al. showed that the influence of spinal sagittal balance on LS and SIA was greater in LS subjects, and Imagama et al. also reported that good spinal sagittal alignment, muscle strength, and 10-m gait speed improved body balance and reduced the risk of falling [10, 24]. Together, these reports demonstrate that global spinal alignment and back muscle strength are related to LS. However, a relationship between global spine sagittal balance and locomotor physical function of each LS risk stage in community-dwelling persons has not been previously reported.

Back muscle weakness may lead to reduced LDH. Strong back muscles contribute to the maintenance of lumbar lordosis and spinal alignment and to better QOL, according to previous studies [3, 26, 29]. Therefore, strengthening and exercises of the back muscles may contribute to the prevention of LS in relatively healthy elderly people. However, back muscle strengthening exercise that is too strenuous may also accelerate LOF as lumbar degeneration by repeated heavy loading, and so mild exercise may be more effective [3, 30], especially in elderly people. A prospective interventional study is needed to evaluate the efficacy of these preventive methods.

In this study, spinal parameters on radiographs were compared with those of Spinal Mouse, and these comparisons validated the accuracy of Spinal Mouse. The thoracic kyphosis angle and lumbar lordosis angle on radiographs were used to confirm the reproducibility of Spinal Mouse measurements. The Spinal Mouse measurements were similar to those of radiographs; although, the measurement levels were different. A significant correlation was observed between radiographs and Spinal Mouse with regard to TKA (*r* = 0.5413, *P* < 0.0001), LLA (*r* = 0.5413, *P* < 0.0001), and global spine sagittal balance (*r* = 0.6253, *P* < 0.0001). This confirmed the reliability of the Spinal Mouse measurements of these angles, and the data could then be used in further analysis. Thus, Spinal Mouse may potentially serve as a reliable alternative to radiographs for assessment of spinal parameters.

There were some limitations in this study. First, only one place and one race of people were analyzed in this study, and the characteristics of people dwelling in the general community were not represented. The subjects were healthy middle-aged and elderly people who lived in a relatively rural area, and many had jobs in agriculture or fishing; therefore, the subjects differed from people in an urban environment. Second, this study was a cross-sectional study. A longitudinal study is necessary to identify the causes of LS. Third, quality of life surveys determined using the Short Form-36 (SF-36) were not performed. The objective measures including mental health should be evaluated in next studies. Beyond these limitations, the findings of this study regarding the relationship between global spine sagittal balance, spinal degenerative change, and LS risk will help improve management of the health of the middle-aged community-dwelling population.

## 5. Conclusion

The subjects with LS have back muscle weakness, degenerative lumbar spine, and kyphotic lumbar alignment. LS subjects also had LBP and global spinal imbalance by anterior spinal inclination and exhibited slower gait speed and narrower stride.

## Figures and Tables

**Table 1:** Subject demographic characteristics among three groups classified by risk for locomotive syndrome.

Variables	All	No risk	Stage 1	Stage 2	*P*
Number of subjects	211	126	56	29	
Age (years)	64.0 ± 10.1	62.8 ± 9.98	65.3 ± 9.79	67.0 ± 10.6	0.1072
Sex (male/female)	89/122	54/72	22/34	13/16	0.8610
Body height (cm)	157.7 ± 8.41	157.4 ± 8.23	158.1 ± 8.47	158.4 ± 9.39	0.9086
Body weight (kg)	58.7 ± 11.6	57.4 ± 11.4	61.9 ± 12.2	59.3 ± 10.4	0.1302
BMI (kg/m^2^)	23.5 ± 3.65	23.0 ± 3.47	24.7 ± 4.32	23.5 ± 2.47	0.0630

Values given are mean ± standard deviation unless otherwise specified. BMI indicates body mass index.

**Table 2:** Comparison of lumbar disc height at each level according to locomotive syndrome risk stage.

Level	All	No risk	Stage 1	Stage 2	*P*
L1–L2 (mm)	9.24 ± 2.52	9.46 ± 2.66	8.96 ± 1.97	8.77 ± 2.71	0.6458
L2–L3 (mm)	9.89 ± 2.60	10.2 ± 2.43	9.50 ± 2.62	9.00 ± 2.97	0.0397^∗^
L3–L4 (mm)	9.92 ± 2.67	10.3 ± 2.56	9.61 ± 2.82	8.91 ± 2.62	0.0338^∗^
L4–L5 (mm)	9.78 ± 2.70	10.3 ± 2.50	8.96 ± 2.82	8.70 ± 2.67	0.0017^∗^
L5–S1 (mm)	8.57 ± 2.61	8.60 ± 2.53	8.42 ± 2.58	8.71 ± 3.01	0.7665

Values given are means ± standard deviations unless otherwise specified. ^∗^Statistically significant.

**Table 3:** Prevalence of lumbar osteophyte formation according to locomotive syndrome risk stage.

Osteophyte formation	All	No risk	Stage 1	Stage 2	*P*
Positive/negative	369/686	203/427	104/176	62/83	
Prevalence	35.0%	32.2%	37.1%	42.8%	0.0380^∗^

^∗^Statistically significant.

## Data Availability

The cohort data used to support the findings of this study are restricted by the Institutional Review Board of Nagoya University Graduate School of Medicine in order to protect the privacy of subjects in Yakumo study.
